# Coexistence of Two Rare Genetic Variants in Canonical and Non-canonical Exons of *SCN5A*: A Potential Source of Misinterpretation

**DOI:** 10.3389/fgene.2021.722291

**Published:** 2021-09-06

**Authors:** Anna G. Shestak, Leonid M. Makarov, Vera N. Komoliatova, Irina V. Kolesnikova, Liubov O. Skorodumova, Edward V. Generozov, Elena V. Zaklyazminskaya

**Affiliations:** ^1^ Russian National Research Center of Surgery Named After B.V. Petrovsky, Moscow, Russia; ^2^Center for Syncope and Cardiac Arrhythmias in Children and Adolescents of the Federal Medical Biological Agency, Moscow, Russia; ^3^Federal Research and Clinical Center of Physical Chemical Medicine of the Federal Medical Biological Agency, Moscow, Russia

**Keywords:** whole exome sequencing, genetic counseling, next generation sequencing, pathogenicity assessment, noncanonical transcripts, *SCN5A* gene

## Abstract

Primary cardiac channelopathies are a group of diseases wherein the role of DNA testing in aiding diagnosis and treatment-based decision-making is gaining increasing attention. However, in some cases, evaluating the pathogenicity of new variants is still challenging. We report an accurate multistage assessment of a rare genetic variant in the *SCN5A* gene using next-generation sequencing (NGS) techniques and Sanger sequencing. Female sportsman (14 years old) underwent genetic counseling and DNA testing due to QT interval prolongation registered during ECG Holter monitoring. Genetic testing of the proband was performed in two independent laboratories. Primary DNA testing was performed by WES using the Ion Proton^TM^ System. Target panel sequencing of 11 genes was performed using PGM Ion Torrent. Search for variants in non-canonical and canonical exons 6 was performed by Sanger sequencing. The cascade familial screening and control re-sequencing were provided for proband with identified genetic variant p.S216L (g.38655290G>A, NM_198056.2:c.647C>T, and rs41276525) in the canonical exon 6 of the *SCN5A* gene after receiving data from another laboratory. Control Sanger and NGS sequencing revealed the absence p.S216L in the canonical exon 6 and confirmed the presence of p.S216L (g.38655522G>A, c.647C>T, and rs201002736) in the non-canonical exon 6 of the *SCN5A* gene. The identified variant was re-interpreted. The non-canonical transcripts of the exon 6 of the *SCN5A* gene is poorly represented in cardiac tissue (gnomAD). The detected variant was found in proband’s healthy mother. The correct interpretation of genetic data requires close cooperation between clinicians and researchers. It can help to avoid financial costs and stress for proband’s and families.

## Introduction

Significant advance has been made in the field of DNA-diagnostic strategies with the development of different massive parallel sequencing methods. These changes are especially pronounced in approaches to perform DNA-based diagnoses of monogenic polyallelic diseases, since it is now possible to simultaneously search for mutations in all candidate genes. Primary cardiac channelopathies are a group of diseases wherein the role of DNA testing in aiding diagnosis and treatment-based decision-making is gaining increasing attention. Although disorders of this class present different electrocardiogram (ECG) phenotypes, they share a high risk of sudden cardiac death (SCD). At present, guidelines prescribed by the American Heart Association, Heart Rhythm Society, European Society of Cardiology, and European Heart Rhythm Association have strongly recommended genetic testing for the diagnosis and treatment of various heart rhythm and conduction disorders (Ackerman et al., 2011; Priori et al., 2013; Al-Khatib et al., 2018).

Although next-generation sequencing (NGS) approaches have their obvious advantages, there are certain difficulties in accurately interpreting massive amounts of genetic data in the clinical context; the greatest being the interpretation of new and rare genetic variants with unknown clinical significance (VUS). Standards and guidelines for the interpretation of genetic findings were first published in 2015 by the American College of Medical Genetics and Genomics (Richards et al., 2015). In 2019, the guidelines were revised to improve the quality of interpretation (Deignan et al., 2019). However, in some cases, evaluating the pathogenicity of new rare variants is still challenging.

In addition, the growing need for diagnostic tests based on NGS is driving the development of new machine learning technologies for solving a wide range of issues in genetic studies, from selecting targets for sequencing to functional annotation of genes or genetic elements (Libbrecht and Noble, 2015). This has significantly reduced the time spent on genetic data processing and has improved accuracy by minimizing human errors in interpretation.

In this article, we report an accurate multistage assessment of a rare genetic variant in the *SCN5A* gene using NGS techniques and direct Sanger sequencing.

## Materials and Methods

Clinical examination and genetic testing were performed in accordance with the principles of the Declaration of Helsinki, and upon the direct request of the patient. Written informed consent from her parents was also obtained. The clinical examination consisted of a general examination, standard ECG at rest and during exercise, and 24-hour Holter monitoring.

Genetic testing was performed in two independent laboratories. Primary DNA testing involved whole exome sequencing (WES) performed on the Ion Proton^TM^ System (Thermo Fisher Scientific, Waltham, MA, United States) using the Ion AmpliSeq^TM^ Exome Kit (Thermo Fisher Scientific). Primary processing of reads was performed using the Ion Proton Software (Thermo Fisher Scientific), and the sequence reads were aligned to the hg19 reference genome using the BWA 0.7.9 software package (Li and Durbin, 2009).

The obtained bam files were filtered using the exome map file, and results were called using the samtools 1.1 package. Finally, variant filtration was completed using the vcf-annotate package and in-house scripts. The vcf file was annotated using the ANNOVAR package (Wang et al., 2010). For WES the coverage >20× in the target area was 88.57%, and >10× was 94.87%.

All called variants for long QT syndrome (LQTS) were filtered by a frequency with a threshold for the maximum allelic contribution of 5.5 × 10^–5^, how it was recommended in “Enhancing rare variant interpretation in inherited arrhythmias through quantitative analysis of consortium disease cohorts and population controls” (Walsh et al., 2021). Then bioinformatic analysis was processed with domestic pipeline. Pathogenicity of all rare variants was assessed using ACMG criteria (Richards et al., 2015), and verified by the latest recommendation for LQTS and Brugada syndrome (BrS) (Richards et al., 2015; Walsh et al., 2021).

An independent target genes panel of 11 genes (*KCNQ1*, *KCNE1*, *KCNE2*, *KCNE3*, *KCNJ2*, *KCNH2*, *SNTA1*, *SCN5A*, *SCN1B*, *SCN3B*, and *SCN4B*) using the Ion Torrent semiconductor sequencing platform (Ion PGM^TM^ System, Thermo Fisher Scientific) was performed. Oligoprimers for this gene panel were designed using the Ion AmpliSeq Designer^®^ online tool (Thermo Fisher Scientific). The reads were preprocessed using Torrent Suite Software 5.6.0, and variant annotation web server Ion Reporter 5.12 (Thermo Fisher Scientific), and 92.15% of the target areas of the genes was covered >20×.

Next-generation sequencing sequencing reads were visualized using the Integrative Genomic Viewer (IGV) tool (Robinson et al., 2011) with hg19 as a reference genome.

We identified a rare genetic variant in the non-canonical exon 6 (chr3: 38655466–38655557) and the canonical exon 6 (chr3: 38655234–38655325) of the *SCN5A* gene. Carrier screening for these rare variants was also performed in the parents using capillary electrophoresis-based Sanger sequencing with two oligoprimer pairs (Supplementary Table 1).

Pathogenicity evaluation for the identified genetic variants was carried out according to the guidelines (Richards et al., 2015; Walsh et al., 2021).

## Results

### Clinical Case

Patient NRF178 was a 14 years old female, competitive athlete (swimming) with 6 years of active sport experience. She had no general health complaints, no history of syncope, no family history of SCD, and she exhibited good exercise tolerance. She was recommended to undergo genetic counseling and testing after an annual cardiac examination.

**FIGURE 1:**
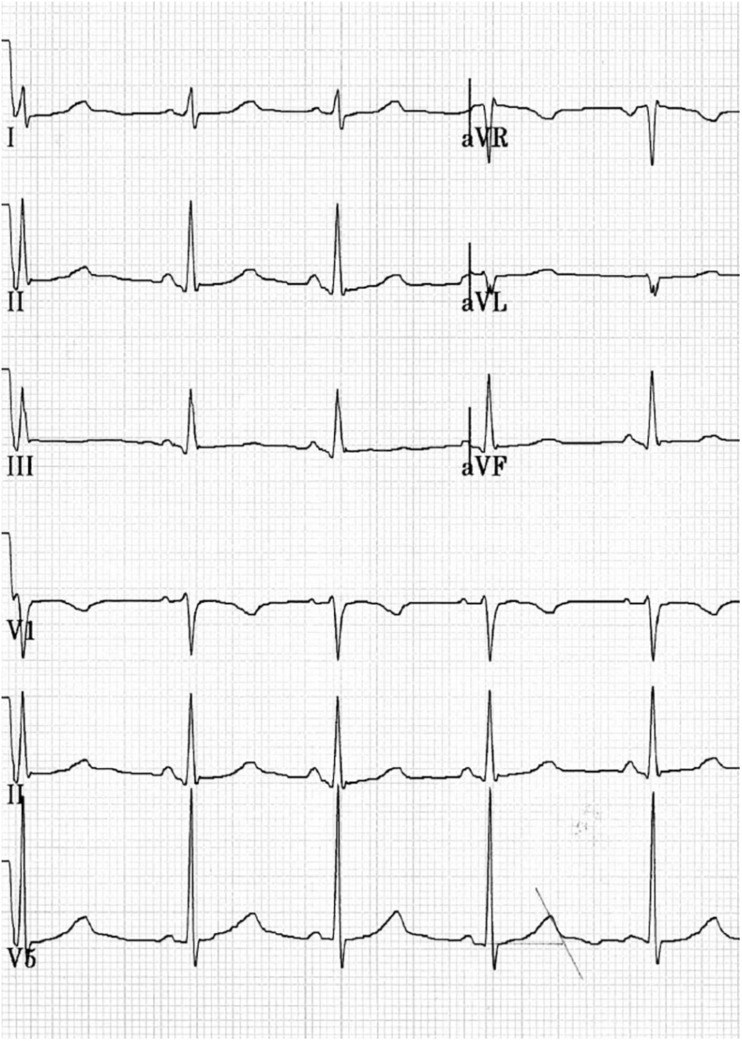
Fragment of the resting ECG, 25 mm/s and 10.0 mm/mV. Female, 14 years old, competitive athlete (swimming). Sinus rhythm with HR 70 bpm, PR 128 ms, QRS 104 ms, and QTc 490–536 ms.

While resting, her ECG showed a sinus rhythm, heart rate of 61 beats/min, QT interval of up to 480–500 ms, and QTc interval of 495 ms. In response to standing up, her ECG showed a sinus rhythm, heart rate of 72 beats/min, QT interval of up to 480 ms, and QTc interval of 535 ms (Figure 1). One month after reduction in training intensity, her ECG showed a sinus rhythm, heart rate of 46 beats/min (bradycardia), with a QT interval of 473 ms, in response to standing up. During the exercise test, at the 4th minute of recovery, the ECG showed a QTc interval of 495 ms. Schwartz’s score was 4.5 points, and the patient was suspected to have LQTS. To confirm this diagnosis, the patient was sent for genetic consultation and subsequent DNA testing.

### DNA Testing Data

Primary DNA testing involving WES revealed a rare genetic variant, g.38655522G>A (c.647C>T, p.S216L, and rs201002736) in the *SCN5A* gene. This variant was identified in a large number of forward and reverse reads and was automatically recognized by the software. It was classified as a VUS (Class III) and was included in the final genetic report with correct genomic coordinates, changes in the coding sequence, and the corresponding amino acid substitution; however, the reference number (rs201002736) and expression profile (minimal expression in the heart) was not mentioned. This final genetic report was directly handed over to the patient’s parents without any genetic counseling. They were then transferred to cardiologists for clinical decision-making. The medical team performed an additional search for information on the genetic variants indicated in the genetic report using “*SCN5A*,” “p.S216L,” and “primary channelopathies” as keywords. The physicians mainly focused on studies wherein the replacement of p.S216L in the canonical exon 6 of the *SCN5A* gene was discussed in a clinical context.

However, given the importance of genetic test results in predicting the patient’s health and for deciding whether to continue her sports career, the family requested a second genetic screening of exon 6 of the *SCN5A* gene in an independent laboratory, without providing the initial results. Based on the parent’s formulated request, targeted analysis of exon 6 of *SCN5A* (canonical isoform, NM_198056.2) was performed by capillary Sanger sequencing, and no genetic abnormalities were reported.

The family declared discrepancies in the results from two genetic laboratories and provided the initial DNA testing report. The next step was to sequence the target panel of 11 genes, including *SCN5A*. The rare genetic variant g.38655522G>A was detected in the non-canonical exon 6 of the *SCN5A* gene (c.647C>T, p.S216L, and rs201002736) by NGS (Supplementary Figure 1) and Sanger direct sequencing (Supplementary Figure 2). Parents were also tested for the presence of this genetic variant. It was also detected in a clinically healthy mother with no signs of cardiovascular disease or QTc interval prolongation in the ECG. According to the Genome Aggregation Database (gnomAD), the minor allele frequency of this variant in the European population is 0.015% (Karczewski et al., 2020). The non-canonical transcripts of exon 6 of the *SCN5A* gene containing this variant were poorly represented in the cardiac tissue (Supplementary Figure 3). This variant was classified as benign (BS1 and BS4 criteria); therefore, it was not included in the final report based on the DNA testing results.

The final report of the repeated genetic tests did not contain any variants. During genetic counseling, family members and cardiologists were informed of the reasons for the apparent discrepancies in the final reports.

The answers for key questions from the family were formulated as follow:

**Q1**. Is there a discrepancy between the results from two genetic laboratories? **A1**. No.
**Q2**. Does variant g.38655522G>A really exists in the proband’s DNA? **A2**. Yes.
**Q3**. Does this mean that the LQTS was confirmed by genetic testing? **A3**. No.
**Q4**. Does this mean that the LQTS was excluded by genetic testing? **A4**. No.
**Q5**. Does this mean that competitive sports are safe or prohibited? **A5**. No/No.
**Q6**. Who and how can they make decisions about sports? **A6**. A shared decision should be made for this ECG-positive genotype-negative patient taking into account QTc dynamics, patient intentions, possible risks, and current recommendations (Pelliccia et al., 2021).

## Discussion

The clinical case presented in this study demonstrates the accurate assessment of a rare genetic variant in the *SCN5A* gene using both NGS and direct Sanger sequencing techniques. Thorough data analysis, additional sequencing, and re-interpretation of the identified substitution helped to avoid any diagnostic errors.

Throughout the multistep examination, the parents repeatedly sought independent consultations from various cardiologists and sports medicine specialists. At least once, the p.S216L variant in the *SCN5A* gene, mentioned in the initial report, was mistaken for a similarly written variant identified in the canonical transcript and accordingly interpreted in the clinical context. At least once, additional tests were assigned to the patient’s relatives and recommendations analogous to those for LQTS were given. Although correctly detected, the variant was incompletely characterized in the report, and the final step of genetic counseling was also skipped. Thus, complete characterization of the variant and genetic counseling could have prevented incautious clinical conclusions. Complete characterization of the variant is also crucial due to the coexistence of two different rare variants with similar descriptive characteristics (Table 1).

**TABLE 1:** Characterization of two genetic variants in the *SCN5A* gene (based on https://gnomad.broadinstitute.org/gene/ENSG00000183873?dataset=gnomad _r2_1).

Gene	*SCN5A*	*SCN5A*
Exon	6 (canonical)	6 (non-canonical)
Genomic coordinate	g.38655290G>A	g.38655522G>A
DNA change	c.647C>T	c.647C>T
Protein	p.S216L	p.S216L
Rs NCBI	rs41276525	rs201002736
Transcript	NM_198056.2	NM_001099404, NM_001099405, NM_001160161, NM_001160160, and NM_001354701
Expression in the heart	High	Trace level
Publication related to this variant	Marangoni et al., 2011	Absent

The p.S216L variant in exon 6 of the *SCN5A* ***canonical transcript*** was first described in a patient with BrS and has been functionally associated with decreased sodium current (Marangoni et al., 2011). S216L is proposed to be an LQTS (Type 3)-causing mutation because of a significant increase in persistent late sodium current and an acceleration in recovery from inactivation. It was also identified in a patient with BrS who presented a significant reduction in persistent sodium current. Therefore, S216L is considered to result in a mixed BrS/LQTS phenotype (Ortiz-Bonnin et al., 2016; Han et al., 2018). In these studies, the p.S216L variant was reported without the transcript number.

The existence of these reports postponed the final diagnosis and decision regarding continuation of her sports career.

A cardiologist usually contextualizes a rare genetic variant in a proband within the “clinical concept” and makes decisions regarding the lifestyle and treatment of the proband. Thus, validation of the initially obtained results by other laboratories was of major importance. In our case, correct interpretation, as well as the format of providing information about the genetic variant would have contributed to avoiding financial costs and stress for the proband’s family.

In his work, Proost (2016) notes that advances in analysis techniques of sequencing data lags behind the progress of NGS technology. As an example, the author mentioned the inability to properly demultiplex data while launching MiSeq (Illumina), which is the first decisive step in data analysis, although the software is built into the sequencer (Proost, 2016). Such difficulties have led to the development of customized pipelines for the annotation and interpretation of genetic variants (Vandeweyer et al., 2014).

The scale of information obtained from high-throughput sequencing requires unification of the detailed interpretations of several genetic variants by the researcher, and this is reflected in modern guidelines (Libbrecht and Noble, 2015; Richards et al., 2015). These guidelines also recommend using the reference genome sequence (RefSeq, Locus Reference Genomic) for precise mapping of the regions to be analyzed during bioinformatic analysis of sequencing data, as well as the use of generally accepted Human Genome Variation Society nomenclature for the accurate description of nucleotide sequence variants. Of note in the reference sequence is the presence of errors associated with the so-called minor reference variants that require correction during bioinformatic analysis (Libbrecht and Noble, 2015).

More recent studies have shown that 12–50% of clinical trial reports significantly contradict clinical reports from other laboratories (Coovadia, 2017). A review has been published showing discrepancies in commonly used databases of genetic variants used to develop clinical genetic tests (Coovadia, 2017). Today, the process of analyzing genetic information requires close cooperation between scientists, geneticists, and bioinformatics researchers. In this multi-stage process, the “human factor” still plays a significant role, meaning that it is a potential source of error.

Studies of diagnostic mistakes across multiple medical specialties indicate that contextual information makes a major contribution to avoiding diagnostic errors and aids in drawing accurate conclusions (Lockhart and Satya-Murti, 2018). The “agnostic” first reading process – a process in which the first reading occurs with a minimum amount of clinical reference information – can be applied to many diagnostic tests. Consequently, research on diagnostic processes shows that it is important to separate the stage of data collection from the stage of their interpretation (Mora et al., 2012; Haber and Haber, 2013). It has been suggested that including medical history and reinterpretation has the potential to reduce errors.

Standard NGS data analysis and automatic gene panel primer design focus on sequencing canonical transcripts, the most clinically significant and/or longest known transcripts of the studied genes (Richards et al., 2015). However, laboratories are required to determine the clinical significance of the variants in all possible transcripts. The identification of genetic variants in non-canonical transcripts and its misinterpretation causes difficulties during subsequent genetic counseling of the proband and their relatives.

Till date, there have been recommendations for establishing contact with patients after re-interpretation of the initial results of genetic testing (Bombard et al., 2019). In this particular case, contact with the proband’s family was immediate in nature.

In conclusion, the present case demonstrates the role of the human behavior in the assessment of the rare genetic variant in the *SCN5A* gene, and this variant has a “twin” in the canonical transcript. Additional sequencing and reinterpretation of the identified replacement allowed for accurate genetic counseling. Today, the correct interpretation of genetic findings requires close collaboration between clinicians, scientists, and bioinformatics researchers. A wide discussion of such diagnostic cases is of great importance for training and professional development of geneticists, cardiologists, and other specialists involved in the process of genetic data interpretation. We believe that the introduction of artificial intelligence-based algorithms for proper interpretation will be a significant step forward in improving the analysis of the collected data and their use in precision medicine. Self-learning algorithms can minimize a “human factor” from the multi-step process of recognition of the candidate genetic variations, variant prioritization, *in silico* prediction mutations effects, and final reporting in the clinical context. Machine-learning methods can also improve significantly recognition of the regulatory sequence patterns, splice sites placed in deep intronic areas, functional annotation of the genes, and co-functional relationship of the gene products. Complementary to the data interpretation, AI-based algorithms might better elicit technical artifacts and other phenomena, such as allelic drop-out, that reduce diagnostic yield of genetic testing (Shestak et al., 2021).

## Data Availability Statement

The original contributions presented in the study are publicly available in NCBI using accession number VCV000925751.2.

## Ethics Statement

The studies involving human participants were reviewed and approved by the local ethics committee. Written informed consent to participate in this study was provided by the participants’ legal guardian/next of kin. Written informed consent was obtained from the individual(s), and minor(s)’ legal guardian/next of kin, for the publication of any potentially identifiable images or data included in this article.

## Author Contributions

AS performed the molecular genetic investigation, data analysis, and drafting the manuscript. LM and VK contributed to the clinical investigation. IK, LS, and EG contributed to the molecular genetic investigation and bioinformatical procession of data. EZ contributed to the genetic counseling, management of the project, and editing and final approval of the manuscript. All authors read, discussed, and approved the manuscript as submitted.

## Conflict of Interest

The authors declare that the research was conducted in the absence of any commercial or financial relationships that could be construed as a potential conflict of interest.

## Publisher’s Note

All claims expressed in this article are solely those of the authors and do not necessarily represent those of their affiliated organizations, or those of the publisher, the editors and the reviewers. Any product that may be evaluated in this article, or claim that may be made by its manufacturer, is not guaranteed or endorsed by the publisher.
