# Supplementary figures and images for: Coexistence of Two Rare Genetic Variants in Canonical and Non-canonical Exons of SCN5A: A Potential Source of Misinterpretation
Source: Front Genet. 2021 Sep 6;12:722291. doi: 10.3389/fgene.2021.722291 (PMC8450431; doi:10.3389/fgene.2021.722291)

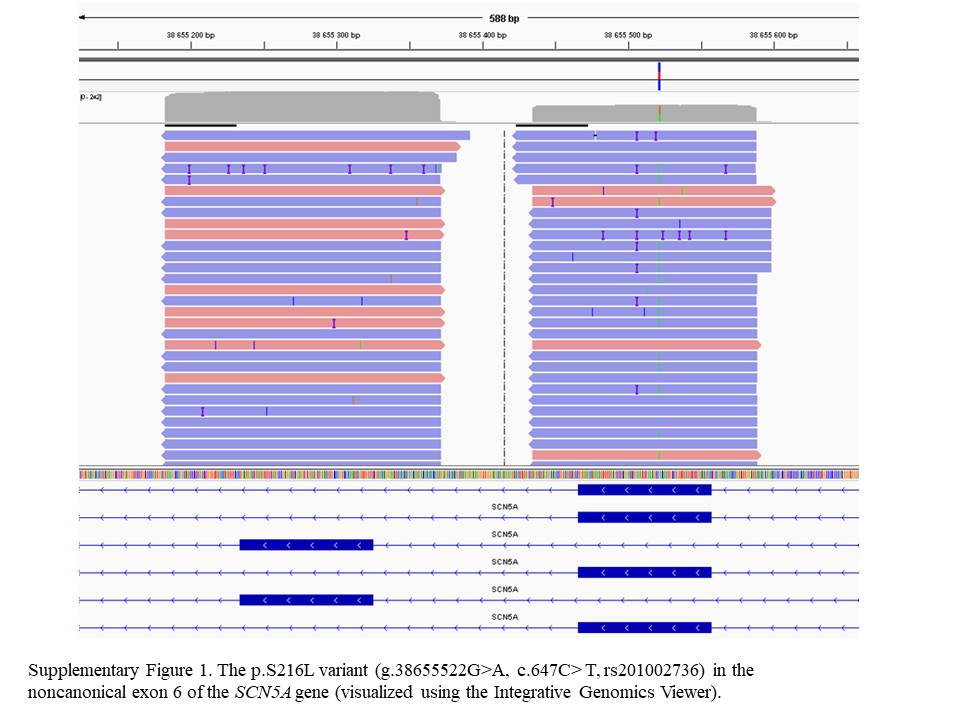

Supplement: Supplementary file 1 [file Image_1.JPEG]

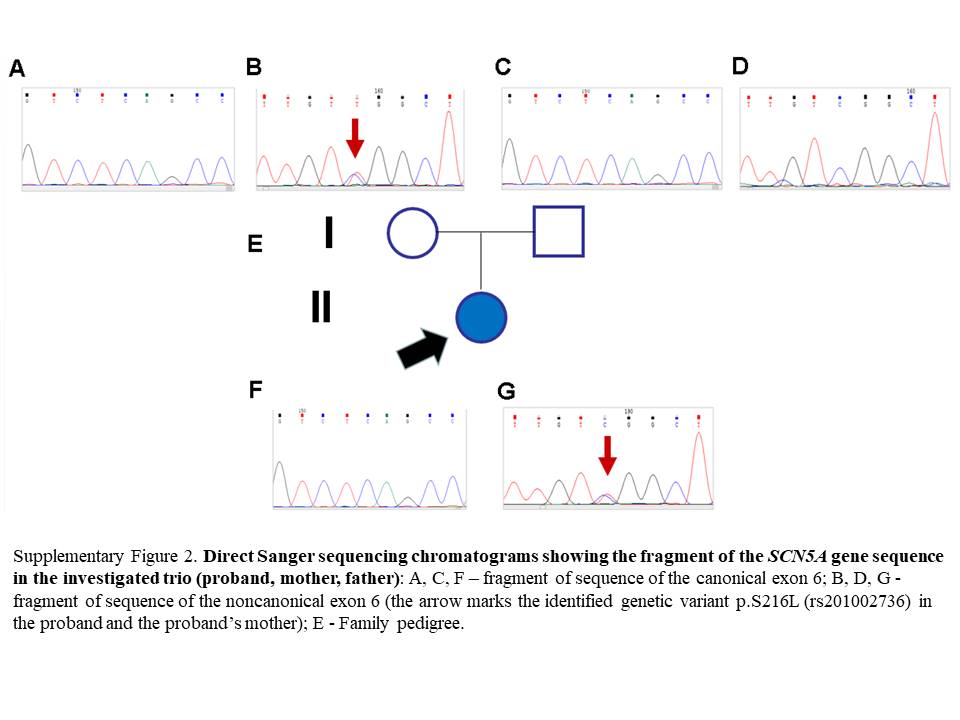

Supplement: Supplementary file 2 [file Image_2.JPEG]

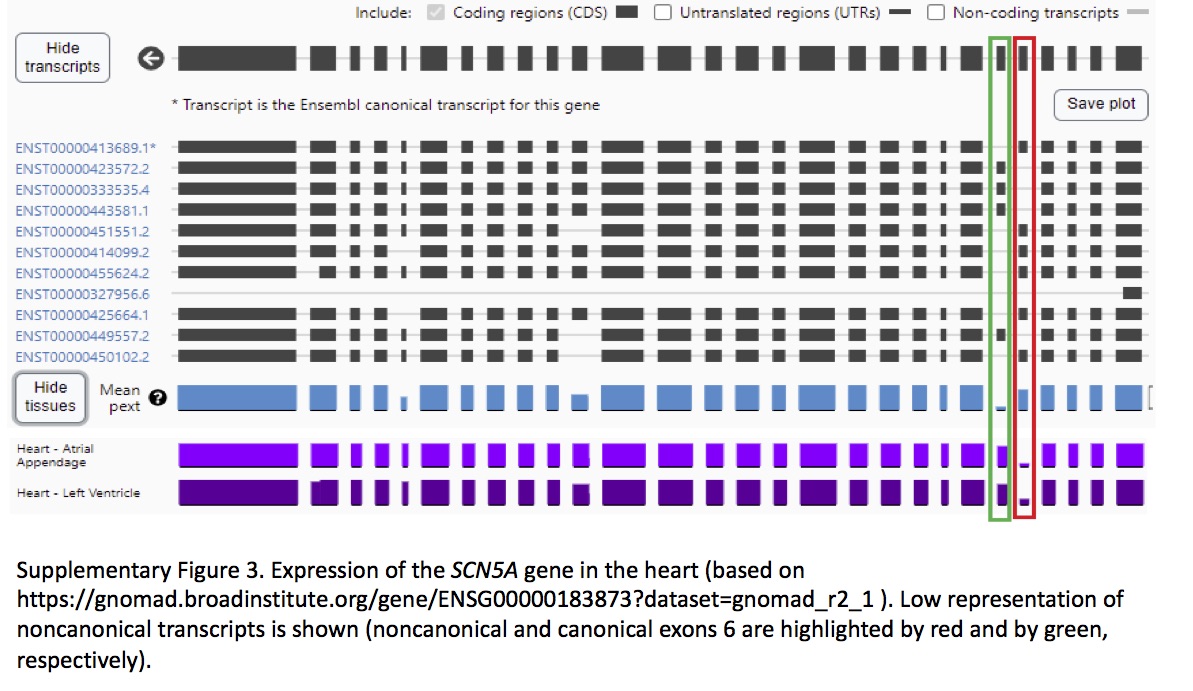

Supplement: Supplementary file 3 [file Image_3.JPEG]
