# Supplementary material for: Coexistence of Two Rare Genetic Variants in Canonical and Non-canonical Exons of SCN5A: A Potential Source of Misinterpretation
Source: Front Genet. 2021 Sep 6;12:722291. doi: 10.3389/fgene.2021.722291 (PMC8450431; doi:10.3389/fgene.2021.722291)
Supplement: Supplementary file 4 [file Table_1.DOCX]

Supplementary Table 1. Oligoprimers sequences used for polymerase chain reaction and direct Sanger sequencing.

| Name | Forward primer | Reverse primer | Amplicon size, bp |
| --- | --- | --- | --- |
| SCN5A-6_nc | TGGGCTATCCACAGCACTGC | GGCCCAGGCATATCCCTCTA | 490 |
| SCN5A-6_c | TATCCCAGGTAAGATGCCCAGGTTTG |  | 286 |
